# Fast, Accurate Assignment of Clinical Diagnoses From Patient Notes by a Large Language Model: Critical Pediatric Pneumonia as a Use Case

**DOI:** 10.1097/CCE.0000000000001350

**Published:** 2025-11-24

**Authors:** Blake Martin, Marisa Payan, Jaime LaVelle, Peter E. DeWitt, Seth Russell, James Mitchell, Sara J. Deakyne Davies, Tellen D. Bennett

**Affiliations:** 1 Section of Critical Care Medicine, Department of Pediatrics, University of Colorado School of Medicine, Aurora, CO.; 2 Department of Biomedical Informatics, University of Colorado School of Medicine, Aurora, CO.; 3 Research Informatics and Data Science, Children’s Hospital Colorado, Aurora, CO.; 4 Division of Pediatric Critical Care, Children’s Hospital Colorado, Aurora, CO.

**Keywords:** bacterial pneumonia, large language models, natural language processing

## Abstract

**OBJECTIVE:**

To determine the accuracy of a custom version of the generative pretrained transformer (GPT)-4o large language model (LLM) in identifying PICU admissions with vs. without bacterial pneumonia using clinical notes.

**DESIGN:**

In this retrospective cohort study, the GPT-4o model was provided guidance on our institution’s pneumonia diagnosis practices through a custom prompt and instructed to analyze PICU provider notes from the first 2 calendar days of PICU admission to identify bacterial pneumonia diagnoses. Diagnoses from the manually curated Virtual Pediatric Systems (VPS) Registry were used as the gold standard.

**SETTING:**

A 48-bed, academic, quaternary care PICU.

**PATIENTS:**

Children 3 months old to 18 years old admitted to the PICU from January 1, 2023, to December 31, 2023.

**INTERVENTIONS:**

None.

**MEASUREMENTS AND MAIN RESULTS:**

GPT-4o analyzed 10,081 notes from 3,317 PICU admissions over 5.0 minutes (mean 0.03 s per note). Of the 3317 study encounters, 481(14.5%) had a VPS admission pneumonia diagnosis. GPT-4o accurately classified 3143 of 3317 (94.8%) encounters. In a post hoc adjudication analysis, a blinded PICU attending reviewed patient charts with VPS-GPT discordant classifications. The GPT-4o classification matched that of the blinded PICU attending in 125 of 174 (71.8%) of such encounters. The most common reason for incorrect classification by GPT-4o was that a pneumonia diagnosis was listed in the initial notes but later rescinded when a different diagnosis was identified.

**CONCLUSIONS:**

The GPT-4o LLM was able to accurately and rapidly identify critically ill children with vs. without bacterial pneumonia. This study suggests similar tools could be developed to automate and accelerate processes typically requiring manual chart review.

**KEY POINTS**

**Question:** Can the generative pretrained transformer (GPT)-4o large language model (LLM), using a customized prompt, accurately identify critically ill children with bacterial pneumonia by analyzing clinical notes?

**Findings:** The GPT-4o tool analyzed 10,081 notes from 3,317 PICU admissions over 5 minutes (~0.03 s per note) with an accuracy of 94.8% compared with manual chart review by a clinical expert.

**Meanings:** GPT-4o accurately identified children with bacterial pneumonia compared with manual expert review. This suggests that LLMs could be used to automate identification of other common clinical diagnoses from patient notes, and, more generally, accelerate processes that have historically required manual chart review.

**RESEARCH IN CONTEXT**

- Given the poor sensitivity of diagnosis and administrative codes, identifying patients with a clinical diagnosis can be challenging.
- Manual chart review to identify patients with clinical diagnoses can be time-consuming and resource-intensive.
- Large language models can rapidly analyze free text, and their use in analyzing clinical notes is an area of active study.

**WHAT THIS STUDY MEANS**

- We leveraged a clinical prompt to create a custom version of the generative pretrained transformer (GPT)-4o model that mimics our unit’s pneumonia diagnosis practices.
- The custom GPT had an accuracy of 94.8% for discriminating critically ill children with vs. without bacterial pneumonia.
- Similar tools could help automate processes requiring manual chart review, enabling researchers to focus their time on more complex tasks.

Effective research and quality improvement (QI) efforts require accurate patient diagnoses and classification into subgroups. For some clinical diagnoses (e.g., bacterial pneumonia, viral bronchiolitis), a single-diagnostic test is rarely available, and administrative/billing codes are often insensitive (1, 2) making identification from the electronic health record (EHR) challenging. Thus, manual chart review is often required for accurate identification of clinical diagnoses. The Virtual Pediatric Systems (VPS, LLC, Los Angeles, CA) Registry, for example, uses trained data abstractors (often an ICU nurse with VPS-specific training) to review patient charts to assign diagnoses, resulting in higher accuracy than administrative/billing codes from the EHR (3). However, such large-scale chart review requires significant time and resources (4), often lagging behind the healthcare encounters of interest by several months.

Within the PICU, bacterial pneumonia is one of the most common serious bacterial infections identified at admission (5, 6) and during subsequent hospitalization (7). It is also a clinical diagnosis that is challenging to identify through traditional methods such as the *International Classification of Diseases* codes (1). Given the high prevalence, many large QI interventions have targeted pneumonia diagnosis and treatment (8–10) and rely on timely, accurate identification of pneumonia cases. As such, new tools are needed to quickly and accurately identify children admitted with bacterial pneumonia.

Large language models (LLMs) are able to rapidly analyze large volumes of free text. One such LLM, the generative pretrained transformer-4o (GPT-4o) model from OpenAI (San Francisco, CA), enables users to create “custom versions” (11) of itself for specific tasks by providing special instructions that the model uses when responding to user input. We hypothesized that a custom GPT would be able to analyze patient notes to accurately classify critically ill children with vs. without bacterial pneumonia.

## METHODS

We included all patients 3 months to 18 years old admitted to a single 48-bed PICU between January 1, 2023, and December 31, 2023. We extracted all notes written by PICU providers (attendings, fellows, residents, and advanced practice providers) from the first 2 calendar days of PICU admission. We constructed a custom GPT prompt reflecting our unit’s bacterial pneumonia diagnosis practices (see **Online Supplement**, https://links.lww.com/CCX/B575). We uploaded the notes to a Health insurance portability and accountability act (HIPAA)-compliant Microsoft (Redmond, WA) Azure cloud computing platform for analysis. The GPT pneumonia tool was instructed to determine the presence vs. absence of a bacterial pneumonia, provide a brief rationale, and provide a confidence score (0–100) for each classification. We leveraged a zero-shot learning approach, meaning that neither patient notes nor outcomes were used to train or fine-tune the model. Instead, we applied the base GPT-4o model directly with a customized prompt.

We calculated the GPT tool’s discrimination using VPS bacterial pneumonia diagnoses as the gold standard and used multivariable logistic regression to identify which preselected variables were associated with correct classification. To determine reproducibility, we ran the model 10 times on the same clinical note input and compared resulting pneumonia diagnoses to the first model run through percent agreement and Cohen’s Kappa. In a post hoc adjudication analysis, a PICU attending physician, blinded to VPS and GPT pneumonia classifications, manually reviewed the charts of PICU encounters with VPS-GPT disagreement. Analyses were performed using R, Version 4.3.3 (R Foundation for Statistical Computing, Vienna, Austria). This study was approved by the Colorado Multiple Institutional Review Board on March 21, 2025, as an addendum to the original study entitled “Predicting Serious Bacterial Infection in the PICU” (protocol number18-2419), both of which were approved for waiver of informed consent given the retrospective, non-interventional nature of the work. All study procedures were conducted in accordance with local ethical standards and the Helsinki Declaration of 1975.

## RESULTS

We extracted 10,081 notes from 3,317 PICU encounters (2,755 unique patients). Of these 3,317 PICU encounters, 481 (14.5%) had a VPS admission pneumonia diagnosis . GPT analysis of the 10,081 notes took a total of 5.0 minutes (averaging 0.03 s/note). Using VPS pneumonia diagnoses as the gold standard, GPT accuracy was 94.8% (3143 correct classifications of 3317 encounters) with pneumonia sensitivity of 84.4% (406 true positives of 481 VPS-positive encounters), specificity 96.5% (2737 true negatives of 2836 VPS-negative encounters), positive predictive value 80.4% (406 true positives of 505 GPT-positive encounters), and negative predictive value 97.3% (2737 true negatives of 2812 GPT-negative encounters). See **Supplemental Results** (https://links.lww.com/CCX/B575) for analysis of the GPT confidence scores.

Over 10 model runs, percent agreement and Cohen’s Kappa when compared with the original model run ranged from 98.2% to 98.8% and 0.931 to 0.953, respectively. In a multivariable logistic regression model, children who were not Hispanic/Latino (as compared with Hispanic/Latino) had higher odds of correct classification by the GPT tool (**Table 1**). While the difference in note length (by number of characters) was statistically significant, the odds ratio 0.9998 (95% CI, 0.9997–0.9999) was not clinically significant.

**TABLE 1.** Characteristics of Study Encounters, Stratified by Correctness of GPT Classification

Variable	GPT Classification Correct, *N* = 3143	GPT Classification Incorrect, *N* = 174	Multivariable Model OR (95% CI)	*p*
Age, median (IQR), yr	3.7 (1.4, 11.5)	5.2 (2.4, 10.5)	1.00 (0.98–1.03)	0.83
Biologic sex				
Female sex, *n* (%)	1,362 (43.3%)	88 (50.6)	Ref	
Male sex, *n* (%)	1,781 (56.7)	86 (49.4)	1.34 (0.98–1.83)	0.06
Race				
White, *n* (%)	2,218 (70.6)	119 (68.4)	Ref	
American Indian/Alaska Native, *n* (%)	23 (0.7)	0 (0.0)	NA	0.98
Asian, *n* (%)	95 (3.0)	5 (2.89)	0.93 (0.40–2.69)	0.87
Black, *n* (%)	284 (9.0)	15 (8.6)	0.96 (0.57–1.74)	0.89
> 1 Race, *n* (%)	114 (3.6)	7 (4.0)	0.80 (0.39–1.93)	0.58
Native Hawaiian/Pacific Islander, *n* (%)	30 (0.95)	2 (1.2)	0.80 (0.23–5.01)	0.76
Other or unknown, *n* (%)	379 (12.1)	26 (14.9)	1.03 (0.64–1.71)	0.90
Ethnicity				
Hispanic or Latino, *n* (%)	1,009 (32.1)	70 (40.2)	Ref	
Not Hispanic or Latino, *n* (%)	2,112 (67.2)	100 (57.5)	1.54 (1.09–2.17)	**0.01**
Unknown, *n* (%)	22 (0.70)	4 (2.3)	0.34 (0.12–1.21)	0.06
Season of admission				
Fall, *n* (%)	793 (25.2)	46 (26.4)	Ref	
Winter, *n* (%)	880 (28.0)	52 (29.9)	0.99 (0.63–1.58)	0.8
Spring, *n* (%)	896 (28.5)	40 (23.0)	1.36 (0.88–2.12)	0.17
Summer, *n* (%)	574 (18.3)	36 (20.7)	0.99 (0.63–1.58)	0.98
Number of notes per patient^a^, median (IQR)	3 (2, 4)	3 (2, 4)	0.87 (0.72–1.06)	0.17
Number of characters per note, median (IQR)	4,314 (3,416, 5,691)	5,084 (3,866, 6,673)	1.00 (1.00, 1.00)^b^	**< 0.001**
Virtual Pediatric Systems pneumonia diagnosis present^c^, *n* (%)	406 (12.9)	75 (43.1)		

GPT = generative pretrained transformer, IQR = interquartile range, OR = odds ratio, PNA = pneumonia, Ref = reference category.

a In the PICU where the study was performed, advanced practice providers and attending providers write separate notes. As such, a patient may have up to six notes written on 1 calendar day if two notes are written at the time of admission, during the daily progress note, and at transfer/ discharge from the PICU for patients who improve quickly and remain in the PICU for < 24 hr.

b Before rounding to the hundredths digit, OR and 95% CI were 0.9998 (0.9997–0.9999).

c Row not included as a variable during multivariable analysis.

Demographic and other variables for study encounters, stratified by GPT-PNA tool accuracy, along with multivariable logistic regression model results comparing GPT-PNA correct vs. incorrect classification encounters.

**Figure 1.**
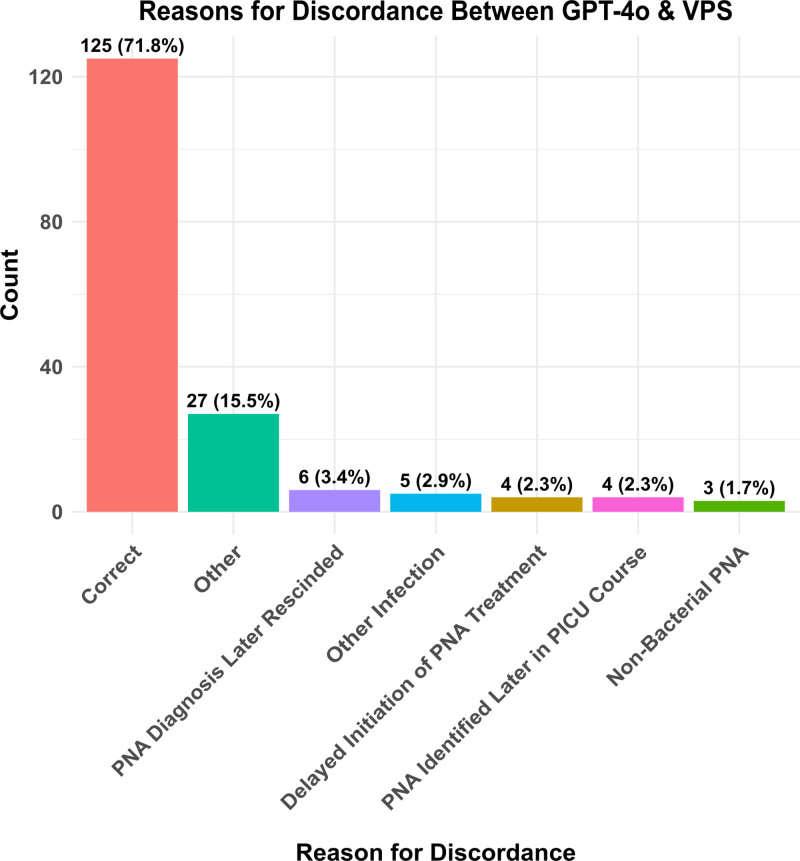
Distribution of the generative pretrained transformer (GPT) pneumonia tool classifications among the 174 Virtual Pediatric Systems (VPS)-GPT discordant encounters (when using manual chart review by a blinded PICU attending physician as the ground truth). “Correct” indicates encounters for which the GPT tool classification matched that of the blinded expert clinician. “Pneumonia (PNA) Diagnosis Later Rescinded” indicates cases for which a pneumonia was initially suspected and treated but later that diagnosis was rescinded and treatment discontinued. “Other Infections” are cases for which the patient experienced respiratory failure and required antibiotics, but a pneumonia was not present. “Delayed Initiation of PNA Treatment” indicates cases for which antibiotics were initially deferred but later initiated when the patient’s condition changed, and a diagnosis of bacterial pneumonia was confirmed. “PNA Identified Later in PICU Course” are cases in which the initial evaluation for bacterial pneumonia was negative but on subsequent evaluation (typically repeat chest radiograph), a PNA was identified. “Non-Bacterial PNA” are cases in which the patient was diagnosed with a non-bacterial (e.g., viral or fungal) pneumonia.

In the adjudication analysis, in which we instead used the blinded PICU attending classification as the gold standard, the GPT tool was correct in 125 of 174 (71.8%) of the VPS-GPT discordant encounters. Among the remaining 49 of 174 (28.2%) of cases (in which the GPT classification did not match that of the PICU attending), the most common reasons for GPT misclassification were: 1) a pneumonia diagnosis was listed in the initial notes but later rescinded when a different diagnosis was identified, 2) the patient received antibiotics and experienced respiratory failure but did not have a bacterial pneumonia, and 3) treatment for pneumonia was not initiated during the first 2 days of PICU admission (i.e., antibiotics were initially deferred but later initiated when the patient’s condition changed and a diagnosis of bacterial pneumonia was felt to be likely enough to warrant antibiotic treatment, **Fig. 1**). Within this same adjudication analysis, we reviewed all 23 of the attending-assigned pneumonia diagnoses that were not identified by the GPT tool. Of these “missed” pneumonia diagnoses, 11 of 23 (48%) were pneumonia diagnoses made by the clinical team after the filing of the PICU provider note on the second day of admission.

## DISCUSSION

In this retrospective cohort study, we designed and validated a custom GPT classifier that accurately identified critically ill children with vs. without bacterial pneumonia with excellent reproducibility. Manual chart review by a blinded expert clinician found that most VPS-GPT discordant cases were correctly classified by the GPT tool. These findings suggest that LLM-based tools may be able to automate the process of retrospective chart review for identification of certain clinical diagnoses, potentially accelerating research and QI efforts and enabling personnel to focus on more complex tasks. Deployment of a small number of similar condition-specific GPT tools could enable automatic detection of the most common PICU diagnoses, which often account for a significant proportion of PICU admissions. In the case of bacterial pneumonia, even if the filing of patient notes lags by several days, this GPT tool could assign pneumonia diagnoses months faster than processes requiring large-scale, manual chart review (such as VPS), allowing for more rapid and frequent iterations on QI initiatives and inclusion of more recently treated patients in retrospective research.

This study has limitations and areas requiring further investigation before broad application. First, this study was performed at a single center. Pneumonia diagnosis and treatment practices and provider clinical documentation patterns may differ between centers. As such, generalizability will need to be tested, and the GPT prompt used may need to be tailored to achieve similar performance at other centers. Similarly, the prompt may need to be updated as clinical note content and patient mix change over time. Second, we found increased odds of GPT accuracy among non-Hispanic/Latino patients compared with Hispanic/Latino patients. Although several studies have identified gender, racial, and ethnic biases when using LLMs (12, 13), the reason for this differential performance (true difference in GPT tool accuracy vs. type I error in the setting of multiple statistical tests) is unclear, and future work should include efforts to understand such discrepancies and ensure equitable performance. Third, clinical notes of the VPS-GPT concordant cases were not manually reviewed by a PICU attending. It is possible that some of these classifications might be deemed incorrect upon review by a PICU attending.

Fourth, although the LLM prompt used reflects our unit’s pneumonia diagnosis practice, we did not explore additional prompt tuning or optimization. Similarly, we evaluated the intrinsic ability of GPT-4o to perform the classification task. Other approaches, such as few-shot learning or model fine-tuning, should be examined in future work. Fifth, this study focused on identification of pneumonia diagnoses from within provider documentation; a multi-modal approach, which incorporates clinical notes, vital signs, and laboratory and imaging results might result in more accurate pneumonia diagnoses. Finally, a sensitivity higher than 84.4% may be required for a particular research or QI project. Given that 48% of the attending-identified pneumonia cases missed by the GPT tool were present in the PICU provider note filed on the third day of PICU admission, it is possible that inclusion of notes from the third day of the PICU encounter may improve sensitivity. Overall, LLM tools may help automate processes that currently require manual patient note review, but further work is needed to ensure generalizability and fairness.

## ACKNOWLEDGMENTS

The authors acknowledge Paul Gervason, U.S. Healthcare, Senior Azure Data and AI Specialist at Microsoft, and Shivang Vora, Senior Cloud Solution Architect for Health and Life Sciences at Microsoft, for their valuable assistance in setting up the online platform on which we designed the custom generative pretrained transformer model used in this study. Microsoft had no role in the study design, data collection, analysis, article preparation, or decision to publish this work. No financial support or funding was provided by Microsoft for this project.

## Supplementary Material


