# Supplementary material for: Fast, Accurate Assignment of Clinical Diagnoses From Patient Notes by a Large Language Model: Critical Pediatric Pneumonia as a Use Case
Source: Crit Care Explor. 2025 Nov 24;7(12):e1350. doi: 10.1097/CCE.0000000000001350 (PMC12647518; doi:10.1097/CCE.0000000000001350)
Supplement: Supplementary file 1 [file cc9-7-e1350-s001.pdf]

Fast, Accurate Assignment of Clinical Diagnoses from Patient Notes by a Large Language Model: Critical Pediatric Pneumonia as a Use Case

Online Supplement

Supplemental Methods

Version 2024-05-13 of OpenAI's GPT-4o model was used to design the GPT-PNA classifier. Below are the instructions (or "prompt") provided to the model for use when analyzing patient notes.

> *"Your task is to serve as a research assistant and perform chart reviews to help with a specific research task. Your job will be to review daily notes from the pediatric intensive care unit (PICU) team to identify, for each patient, whether or not the PICU team diagnosed that patient with a bacterial pneumonia. For each patient, you will review one to six daily notes from the PICU provider team. You will review each patient's notes and make a determination whether or not that patient was deemed to have a bacterial pneumonia. To help you with this task, please follow these additional instructions:*
>
> *1. Physicians often use abbreviations in their notes. Here are a few definitions which will help you in your task:*
>
> *a. CAP stands for community acquired pneumonia and is used to denote a bacterial pneumonia (unless otherwise stated) that was acquired outside of the hospital.*
>
> *b. HAP stands for hospital acquired pneumonia. This is also used to denote a bacterial pneumonia (unless otherwise stated); specifically, one that was acquired in the hospital.*
>
> *c. PNA stands for pneumonia. It is often used when a patient has a bacterial pneumonia, however context is important as viral and fungal pneumonias (which could be denoted 'viral PNA' or 'fungal PNA') are also common conditions in the PICU and would not count toward a diagnosis of bacterial pneumonia for your task. In the absence of the note specifying a fungal or viral process, the acronym of PNA should be assumed to reflect a bacterial pneumonia.*
>
> *d. LRTI stands for lower respiratory tract infection. This is most often used to denote a viral infection. If a bacterial process is not specifically mentioned, LRTI should be assumed to reference a viral process.*
>
> *e. ARF usually stands for acute respiratory failure. Though all patients in the PICU with a bacterial pneumonia will have acute respiratory failure, many patients with acute*

respiratory failure will not have a bacterial pneumonia. Other conditions, especially viral bronchiolitis and viral pneumonitis, can cause acute respiratory failure.

f. VAP stands for ventilator associated pneumonia. These always refer to bacterial pneumonias unless otherwise stated.

2. The conditions of bronchiolitis and pneumonitis are nearly always in reference to non-bacterial processes such as viral infections. Patients with viral bronchiolitis or viral pneumonitis can also have superimposed bacterial pneumonias, but if a bacterial pneumonia is not being treated with antibiotics nor specifically diagnosed elsewhere within the note text, then that patient with bronchiolitis or pneumonitis should be assumed to have a viral infection only (and not a bacterial pneumonia).

3. Many of the notes that you will be analyzing will not be explicit with regard to a pneumonia diagnosis. Any of the following scenarios could be used to describe a child with bacterial pneumonia and should be classified as a bacterial pneumonia:

a. An attending may say that a patient has a 'consolidation' in their chest (or on their chest X-ray). If a consolidation is noted and the patient is being treated with antibiotics, then this patient should be classified as having a bacterial pneumonia.

b. The attending may note that the patient has sepsis with a respiratory source. Since sepsis is nearly always bacterial when referenced in PICU provider notes, this should be classified as a bacterial pneumonia.

c. An attending may say that that one or both of the patient's lungs are 'whited out' or 'consolidated.' If such a description is present and the patient is being treated with antibiotics, then this patient should be classified as having a bacterial pneumonia unless a separate reason is provided for the pulmonary findings.

d. The note may state that the patient has a serious bacterial infection (SBI) causing respiratory failure: this is a bacterial pneumonia.

e. The note may state that the patient is being treated for an aspiration pneumonia or aspiration pneumonitis, which should be classified as a bacterial pneumonia. Further, if the note describes that aspiration has contributed to respiratory failure and that patient is being treated with antibiotics, then that patient should be considered to have a bacterial pneumonia.

f. If a patient has a pleural effusion, does not have a separate clear bacterial source of infection, and is being treated with antibiotics, then this patient should be classified as having a bacterial pneumonia.

4. The following conditions should be counted as bacterial pneumonias, unless otherwise stated, as they are essentially always associated with or caused by a bacterial pneumonia:

a. Pulmonary abscess, lung abscess, or pleural abscess

b. Pulmonary bacterial infection

c. Pleural or pulmonary empyema

d. Complicated pneumonia

e. Necrotizing pneumonia

5. You will usually be reviewing two PICU progress notes (though the number of notes per PICU encounter can range from 1 note to 6 notes). Sometimes the notes may conflict with respect to a diagnosis of bacterial pneumonia. For your purposes, if 2 or more notes conflict, the contents of the last note (e.g. the 6th note if there are 6 notes) should be used to determine presence vs. absence of bacterial pneumonia.

The input data will be provided to you in in a structured format, with the text from all of the patient's notes given to you as a single string. Within that string, there will be text to identify the beginning and end of each note with reference to how many notes there are for that patient. For example, the second note for a patient with three notes will begin with "Note 2 of 3: " followed by that note's text. The end of each individual note will be denoted by the string " |End of Note| " signaling the end of that note.

For each patient, your output should include, in csv format, the following 3 outputs:

a. Your classification for the patient's note(s) as either 'bacterial pneumonia present' or 'bacterial pneumonia not present', to indicate if you identify a bacterial pneumonia or not, respectively.

b. A 1-3 sentence summary of how you came to your conclusion (contained within quotation marks).

c. A confidence score in the range of 0-100 (inclusive) indicating how confident you are that the patient has a bacterial pneumonia, where 0 indicates minimal confidence and 100 represents complete confidence."

Supplemental Results

Supplemental Table 1. GPT Pneumonia Classifier Performance by Confidence Score

Confidence Score Range	# of Patients	Accuracy	Sensitivity	Specificity	PPV	NPV
1-20	1	1	1	NA	NA	NA
21-40	0	NA	NA	NA	NA	NA
41-60	1	1	1	NA	NA	NA
61-80	40	0.6	0.85	0.08	0.66	0.2
81-100	3,275	0.95	0.97	0.87	0.98	0.81

Performance metrics of the GPT pneumonia classifier, stratified by confidence score quintile. Abbreviations: GPT = generative, pretrained transformer; NPV = negative predictive value; PPV = positive predictive value

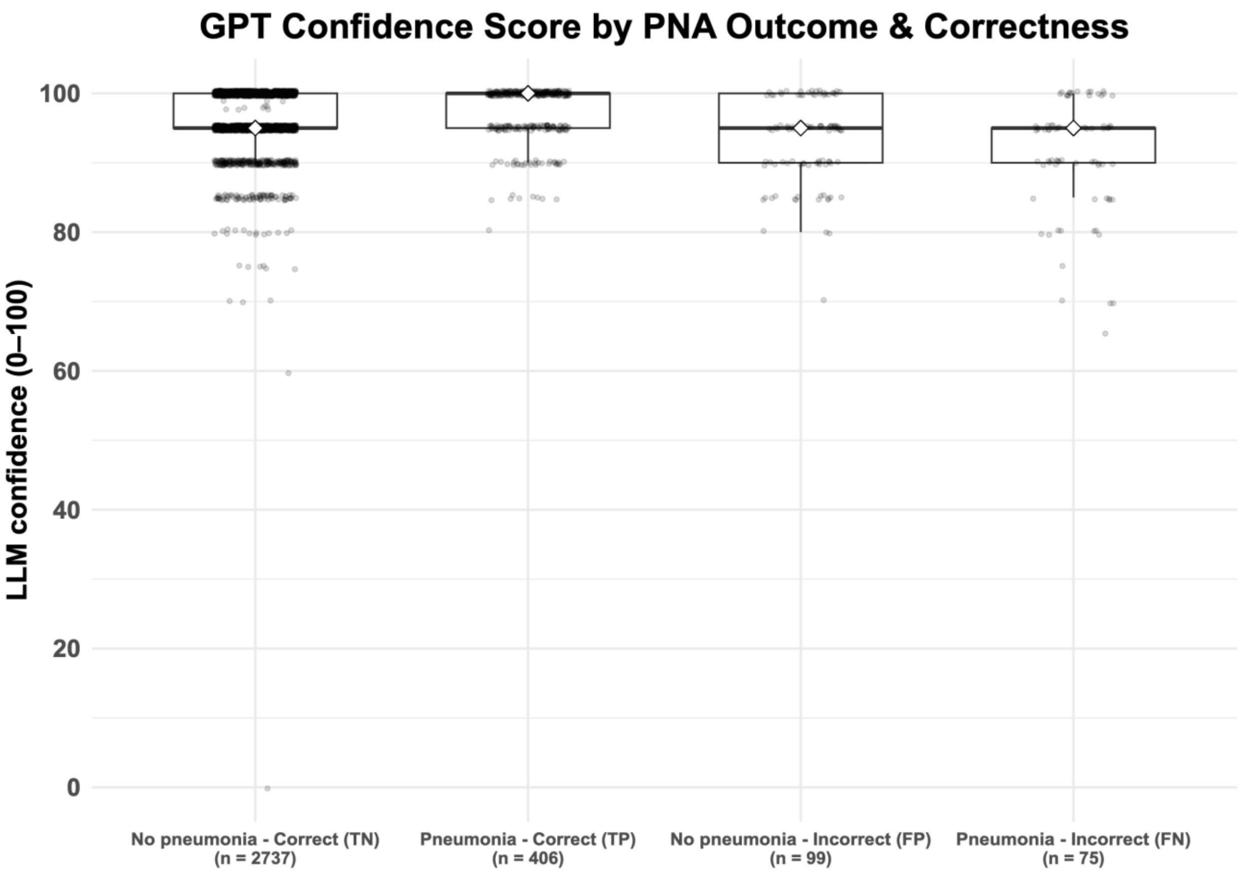

Supplemental Figure 1. Distribution of confidence scores generated by the custom GPT large language model, stratified by true presence vs. absence of pneumonia as well as correctness Abbreviations: FN = false negative; FP = false positive; GPT = generative, pretrained transformer; LLM = large language model; PNA = pneumonia; TN = true negative, TP = true positive
